# Supplementary material for: Two founder variants account for over 90% of pathogenic BRCA alleles in the Orkney and Shetland Isles in Scotland
Source: Eur J Hum Genet. 2024 Oct 22;32(12):1624–31. doi: 10.1038/s41431-024-01704-w (PMC11607322; doi:10.1038/s41431-024-01704-w)
Supplement: Supplementary file 1 — Supplemental Table 1 [file 41431_2024_1704_MOESM1_ESM.pdf]

gene	c.	p.	rsid	GRCh38	consequence	LOF	notes	ClinVar
BRCA1	c.5207T>C	p.Val1736Ala	rs45553935	17:43057122:A:G	missense	0	founder variant	https://www.ncbi.nlm.nih.gov/clinvar/variation/37648/
BRCA2	c.517-2A>G	-	rs81002858	13:32326497:A:G	splice acceptor	1	founder variant	https://www.ncbi.nlm.nih.gov/clinvar/variation/51801/
BRCA2	c.5073dupA	p.Trp1692Metfs	rs80359480	13:32339421:C:CA	frameshift	1	singleton	https://www.ncbi.nlm.nih.gov/clinvar/variation/37943/
BRCA2	c.6275_6276del	p.Leu2092fs	rs11571658	13:32340629:CTT:C	frameshift	1	singleton inherited from Scottish Mainland parent	https://www.ncbi.nlm.nih.gov/clinvar/variation/9318/
BRCA2	c.6814del	p.Arg2272fs	rs397507885	13:32341164:GA:G	frameshift	1	singleton	https://www.ncbi.nlm.nih.gov/clinvar/variation/52196/
BRCA2	c.8167G>C	p.Asp2723His	rs41293511	13:32363369:G:C	missense	0	singleton inherited from Scottish Mainland parent	https://www.ncbi.nlm.nih.gov/clinvar/variation/52515/

Supplementary Table 1 Actionable BRCA1/2 variants in Orkney and Shetland. Pathogenic or likely pathogenic variants in BRCA1 or BRCA2 among 4198 exomes from the Northern Isles.
All six variants have 3* status in ClinVar as pathogenic and were reviewed by an expert panel, all are autosomal dominant.
c. gives the coding DNA position and change; p, the protein position and change; rsid, reference SNP cluster ID; GRCh38, Genome Reference Consortium human build 38; LOF, loss of function.
