# Supplementary material for: Two founder variants account for over 90% of pathogenic BRCA alleles in the Orkney and Shetland Isles in Scotland
Source: Eur J Hum Genet. 2024 Oct 22;32(12):1624–31. doi: 10.1038/s41431-024-01704-w (PMC11607322; doi:10.1038/s41431-024-01704-w)
Supplement: Supplementary file 3 — Details of Gene and Sequence Variants Studied [file 41431_2024_1704_MOESM3_ESM.docx]

**Details of Gene and Sequence Variants Studied**

*Gene Names*

BRCA1

BRCA2

KCNH2

TP53

PALB2

STK11

PTEN

APC

RET

MAX

TMEM127

BMPR1A

SMAD4

MLH1

MLH2

MSH6

PMS2

MEN1

MUTYH

NF2

SDHD

SDHAF2

SDHC

SDHB

RB1

VHL

WT1

ATM

CHEK2

*Sequence Variants*

ATM c.7271T>G p.Val2424Gly rs28904921

BRCA1 c.5207T>C p.Val1736Ala rs45553935

BRCA1 2800delAA p.Lys894fs

BRCA1 c.68_69del 185delAG rs80357914

BRCA1 c.5266dup 5382insC rs80357906

BRCA2 c.517-2A>G (also called IVS6-2A>G) rs81002858

BRCA2 c.6275_6276del p.Leu2092fs (also called 6503delTT) rs11571658

BRCA2 c.771_775 p.Asn257LysfsTer17 (also called del 999del5) rs80359671

BRCA2 c.5946del 6174delT rs80359550

CHEK2 c.1100del rs555607708
